# Pepper CabZIP63 acts as a positive regulator during *Ralstonia solanacearum* or high temperature–high humidity challenge in a positive feedback loop with CaWRKY40

**DOI:** 10.1093/jxb/erw069

**Published:** 2016-03-01

**Authors:** Lei Shen, Zhiqin Liu, Sheng Yang, Tong Yang, Jiaqi Liang, Jiayu Wen, Yanyan Liu, Jiazhi Li, Lanping Shi, Qian Tang, Wei Shi, Jiong Hu, Cailing Liu, Yangwen Zhang, Wei Lin, Rongzhang Wang, Huanxin Yu, Shaoliang Mou, Ansar Hussain, Wei Cheng, Hanyang Cai, Li He, Deyi Guan, Yang Wu, Shuilin He

**Affiliations:** ^1^National Education Minister, Key Laboratory of Plant Genetic Improvement and Comprehensive Utilization Fujian Agriculture and Forestry University, Fuzhou, Fujian 350002, PR China; ^2^College of Crop Science, Fujian Agriculture and Forestry University, Fuzhou, Fujian 350002, PR China; ^3^College of Life Science, Fujian Agriculture and Forestry University, Fuzhou, Fujian 350002, PR China; ^4^College of Life Science, Jinggang Shan University, Ji’an, Jiangxi 343000, PR China

**Keywords:** CabZIP63, CaWRKY40, high temperature–high humidity, pepper, *Ralstonia solanacearum*, transcription factors.

## Abstract

CaWRKY40 is known to act as a positive regulator in the response of pepper (*Capsicum annuum*) to *Ralstonia solanacearum* inoculation (RSI) or high temperature–high humidity (HTHH), but the underlying mechanism remains elusive. Herein, we report that CabZIP63, a pepper bZIP family member, participates in this process by regulating the expression of *CaWRKY40*. CabZIP63 was found to localize in the nuclei, be up-regulated by RSI or HTHH, bind to promoters of both *CabZIP63* (*pCabZIP63*) and *CaWRKY40* (*pCaWRKY40*), and activate *pCabZIP63*- and *pCaWRKY40*-driven β-glucuronidase expression in a C- or G-box-dependent manner. Silencing of *CabZIP63* by virus-induced gene silencing (VIGS) in pepper plants significantly attenuated their resistance to RSI and tolerance to HTHH, accompanied by down-regulation of immunity- or thermotolerance-associated *CaPR1*, *CaNPR1*, *CaDEF1*, and *CaHSP24*. Hypersensitive response-mediated cell death and expression of the tested immunity- and thermotolerance-associated marker genes were induced by transient overexpression (TOE) of *CabZIP63*, but decreased by that of *CabZIP63-SRDX*. Additionally, binding of CabZIP63 to *pCaWRKY40* was up-regulated by RSI or HTHH, and the transcript level of *CaWRKY40* and binding of CaWRKY40 to the promoters of *CaPR1*, *CaNPR1*, *CaDEF1* and *CaHSP24* were up-regulated by TOE of *CabZIP63.* On the other hand, *CabZIP63* was also up-regulated transcriptionally by TOE of *CaWRKY40*. The data suggest collectively that CabZIP63 directly or indirectly regulates the expression of *CaWRKY40* at both the transcriptional and post-transcriptional level, forming a positive feedback loop with CaWRKY40 during pepper’s response to RSI or HTHH. Altogether, our data will help to elucidate the underlying mechanism of crosstalk between pepper’s response to RSI and HTHH.

## Highlight

CabZIP63, indirectly activated by CaWRKY40, positively modulates transcription of *CabZIP63* and *CaWRKY40*, enhances the binding of CaWRKY40 to its target promoters, and, therefore, increases resistance to *Ralstonia solanacearum* and thermotolerance.

## Introduction

Accumulating evidence indicates that extensive transcriptional reprogramming of multiple genes is generally activated in plants by exposure to various biotic or abiotic stresses individually or simultaneously, which leads to an appropriate downstream defense response (Atkinson and Urwin, 2012; Prasch and Sonnewald, 2013; Rasmussen *et al.*, 2013; Zhu *et al.*, 2013; Cao *et al.*, 2014; Sewelam *et al.*, 2014). Transcription factors (TFs) are extensively involved and act as regulators in the transcriptional reprogramming through recognizing and binding their cognate *cis*-elements in promoters of clusters of target genes, and the TFs themselves are also orchestrated by multiple upstream signaling components, constituting a complicated TF network modulating the expression of a huge number of responding genes (Atkinson and Urwin, 2012; Lindemose *et al.*, 2013; Schweizer *et al.*, 2013; Rabara *et al.*, 2014). A better understanding of the mechanism underlying the network will benefit genetic engineering to improve crop tolerance/resistance to various stresses.

WRKYs are one of the largest plant-specific TF families, characterized by their conserved WRKY domain, which recognizes and bind to the cognate W-box (TTGACC) enriched in the promoters of their target genes and transcriptionally modify their expression (Eulgem *et al.*, 2000; Ulker and Somssich, 2004; Wu *et al.*, 2005). Besides their role in plant growth and development, the majority of WRKY family members have also been implicated in plant responses to different biotic stresses, including pathogens (Cheng *et al.*, 2015), herbivores (Skibbe *et al.*, 2008), and viruses (Chen *et al.*, 2013), abiotic stresses including drought (Jiang *et al.*, 2012; Niu *et al.*, 2012; Luo *et al.*, 2013), heat (Li *et al.*, 2011), salt (Niu *et al.*, 2012), and freezing (Niu *et al.*, 2012), and phytohormone signaling including abscisic acid (ABA) (Rushton *et al.*, 2012), salicylic acid (SA) (Knoth *et al.*, 2007; Shimono *et al.*, 2007), jasmonic acid (JA) (Gao *et al.*, 2011), and ethylene (ET) (Chen *et al.*, 2013). These WRKY genes are generally transcriptionally up- or down-regulated by stresses, acting as activators or repressors in the response of plants to these stresses. New findings demonstrate that the functions of WRKY TFs and the underlying mechanisms are complicated; the WRKY genes involved in plant response to different stresses generally exhibit transcriptionally inducible expression, and multiple *cis*-elements including the W-box are consistently present in their promoters, suggesting extensive autoregulation and cross-regulation by WRKY itself and various upstream TFs and other signaling components (Eulgem and Somssich, 2007; Rushton *et al.*, 2010; Llorca *et al.*, 2014). However, knowledge of the possible transcriptional regulators of WRKY TFs and how they operate is very limited so far. In addition, a single WRKY TF may be modified transcriptionally by multiple stresses, and these TFs are involved in several seemingly disparate processes (Zhang *et al.*, 2008; Rushton *et al.*, 2010; Chen *et al.*, 2012; Yu *et al.*, 2012; Zhu *et al.*, 2013). These findings indicate that WRKY proteins might act as convergent nodes in the crosstalk between or among different biological processes, which provides great potential for plants, allowings fine-tuning of specific biological processes and co-ordination of multiple biological processes (Schweiger *et al.*, 2014). However, the underlying mechanism remains largely unknown.

Another large TF family are the basic leucine zippers (bZIPs); the members of this family are characterized by a 40–80 amino acid conserved bZIP domain, which possesses a basic region that binds DNA and an adjacent Leu (leucine) zipper region that mediates protein dimerization (Hurst, 1995). Through preferential binding of the basic region to DNA sequences with an ACGT core *cis*-element, in particular the G-box (CACGTG), C-box (GACGTC), and A-box (TACGTA) (Izawa *et al.*, 1993; Foster *et al.*, 1994), bZIPs transcriptionally modify the expression of a vast array of target genes and play important roles in diverse physiological processes in plant growth, development, and responses to abiotic stresses, such as salt, drought (Lee *et al.*, 2006), and nitrogen (Lopez-Berges *et al.*, 2010), biotic stresses (Pontier *et al.*, 2001; Kim and Delaney, 2002; Zander *et al.*, 2010; Zhong *et al.*, 2015), as well as signaling mediated by phytohormones such as SA, JA, ET, and ABA; some members act as negative regulators and some members act as positive regulators (Pontier *et al.*, 2001; Zander *et al.*, 2010, 2012). As well as being regulated at the transcriptional level, bZIPs are also modified at the post-translational level via the formation of heterodimers or homodimers (Llorca *et al.*, 2014). In Arabidopsis, some bZIPs such as the TGA family of TFs participate in SA signaling regulation by interacting with NPR1 (Despres *et al.*, 2000; Kim and Delaney, 2002; Shearer *et al.*, 2012), but some clade I TGA TFs were found to act in an NPR1-independent manner (Shearer *et al.*, 2012). As mentioned above, although bZIP and WRKY are involved in similar biological processes and some of these processes overlap, information on the functional relationship between bZIPs and WRKYs is very limited.

Pepper (*Capsicum annuum*) is a vegetable of great economic importance worldwide and also a typical member of the *Solanaceae*, with various soil-borne diseases, which generally cause heavy loss in pepper production, especially under high temperature–high humidity (HTHH) conditions. Unraveling the molecular mechanism underlying the pathogen response under HTHH will enable us to explore and manipulate crucial regulatory nodes in order to enhance disease resistance under HTHH conditions. Previously, we found that CaWRKY40, which is transcriptionally up-regulated both by high temperature under high humidity and by *Ralstonia solanacearum* inoculation (RSI), acts as a positive regulator in the response of pepper to *R. solanacearum* infection and in thermotolerance under high humidity (Dang *et al.*, 2013). CaWRKY6, another member of the pepper WRKY family, also acts as a positive regulator in the same process by binding to the promoter of *CaWRKY40* and directly activating the transcriptional expression of *CaWRKY40* (Cai *et al.*, 2015). In the present study, a positive clone was isolated by the yeast one-hybrid system from a cDNA library of pepper using the promoter of *CaWRKY40* as bait. The positive clone turned out to be *CabZIP63*, which is up-regulated by RSI or HTHH, and acts as a positive regulator in the response of pepper to RSI or HTHH by acting directly upstream of CaWRKY40, forming a positive feedback loop with CaWRKY40 during pepper’s response to RSI or HTHH.

## Materials and methods

### Plant materials and growth conditions

Seeds of pepper (*C. annuum*) or inbred lines Zunla-1, GZ03, and XJ116, and *Nicotiana benthamiana*, provided by the pepper breeding group in Fujian Agriculture and Forestry University (Fuzhou, China), were sown in a soil mix [peat moss:perlite, 2:1 (v/v)] in plastic pots and placed in a growth room under at 25 °C, 60–70 µmol photons m^−2^ s^−1^, a relative humidity of 70%, and a 16h light/8h dark photoperiod.

### Pathogens and inoculation procedures

*Ralstonia solanacearum* strain FJC100301 was isolated previously in our lab and amplified according to the method of Dang *et al.* (2013). The bacterial cell solution used for RSI of pepper plants for functional characterization of *CabZIP63* was diluted to 10^8^ cfu ml^−1^ (OD_600_=0.8) with 10mM MgCl_2_. Pepper plants were inoculated by infiltrating 10ml of the resulting *R. solanacearum* suspension into the third leaves of pepper plants at the eight-leaf stage from the apical meristem using a syringe without a needle, and mock inoculation was with sterile 10mM MgCl_2_. The leaves were harvested at the indicated time points for the preparation of RNA or for other assays such as trypan blue and 3,3′-diaminobenzidine (DAB) staining.

The virulence of *R. solanacearum* strain FJC100301 was assayed by irrigation of injured roots using two inbred pepper lines, GZ03 and XJ116. Each pot containing one plant at the six- to eight-leaf stage with its root injured was irrigated with 5ml of FJC100301 suspension containing 1×10^5^ cells ml^–1^, and then the pots were kept in a growth room at 28 °C with soil moisture at >90%. The disease indexes of the plants were evaluated at 7 days post-inoculation (dpi) following the standards published by the Ministry of Agriculture of the People’s Republic of China (Supplementary Table S1 at *JXB* online).

### Treatment of plants with exogenous hormones and HTHH

Pepper plants at the four-leaf stage were sprayed with 1mM SA, 100 µM methyl jasmonate (MeJA), 100 µM ABA, or 100 µM ethephon (ETH). Mock-treated plants were sprayed with the corresponding solvent or sterile ddH_2_O. For HTHH treatment, pepper plants at the eight-leaf stage were kept under high temperature (38 °C) and 90% humidity or normal temperature (25 °C) and 50% humidity; to ensure that cell death under HTHH did not result from photo-oxidative stress, plants were put in the dark before harvesting for further analysis.

### Yeast one-hybrid screening

Screening was performed using the Matchmaker™ one-hybrid system (Clontech, Palo Alto, CA, USA). To make a target–reporter construct, a fragment in the promoter of *CaWRKY40* containing a C-box and a G-box (from –1889 to –1551 where the translation start codon of *CaWRKY40* was set as +1) was inserted into the *Kpn*I and *Xho*I sites of plasmid pAbAi . The recombinant vector was sequenced and transformed into the yeast strain Y1HGold (Clontech) by polyethylene glycol (PEG)-mediated transformation to generate the yeast bait strain. The pepper Matchmake™ cDNA expression library (Zunla-1) constructed previously in our lab was used for screening for positive clones interacting with the C- and G-box-containing *pCaWRKY40* according to the protocol provided by the Matchmaker™ one-hybrid system (Clontech). A 15ml yeast culture was transformed using 3 μg of the cDNA and plated on synthetic minimal medium containing 400ng ml^–1^ AbA^r^ (Aureobasidin A), but lacking uracil. After incubation at 30 °C for 3 d, the colonies were transferred to filter paper and tested for β-galactosidase activity. Plasmids were extracted from the positive yeast colonies, amplified in *Escherichia coli* cells, and purified for sequencing. The sequences of the positive clones were used as a query to search the genome sequence banks (http://peppersequence.genomics.cn/page/species/index.jsp), and its corresponding promoter sequence was determined.

### Vector construction

For vector construction, a Gateway cloning technique (Invitrogen, Carlsbad, CA, USA) and a series of Gateway-compatible destination vectors were employed. The full-length cDNA of *CabZIP63* and *CaWRKY40*, and the promoter region of *CabZIP63* (2000bp upstream of ATG, *pCabZIP63*), were initially amplified by PCR with their corresponding specific primer pair (Supplementary Table S2) flanked with attB for Gateway cloning and GXL DNA polymerase (Takara, Osaka, Japan), and confirmed by sequencing. The full-length cDNAs were cloned into the entry vector pDONR207 by BP reaction, and then into destination vectors such as pMDC83, pK7WG2, and pEarleyGate201 by LR reaction for subcellular localization, transient overexpression, and ChIP analysis, respectively. *pCabZIP63* was cloned into the pMDC163 destination vector for expression assay of the *pCabZIP63*-driven β-glucuronidase (GUS) reporter gene in pepper plants. To construct the vectors for virus-induced gene silencing (VIGS), a fragmens of ~229bp in length in the 3′-untranslated region (UTR) of *CabZIP63* or *CaWRKY40* was amplified by PCR with a specific primer pair, and was cloned sequentially into entry vector pDONR207 and the destination vector, the PYL279 VIGS vector, using the Gateway cloning technique (Invitrogen) similarly to as described above. For the vector construction for the dominant repressor version of *CabZIP63* or *CaWRKY40*, the EAR repression domain (SRDX) (Hiratsu *et al.*, 2003) was fused to the 3′ terminus of *CabZIP63* or *CaWRKY40* by PCR using the primers modified according to the sequence of the SRDX domain (5′-CTCGATCTGGATCTAGAACT CCGTTTGGGTTTCGCT-3′). Subsequently the *CabZIP63-SRDX* or *CaWRKY40-SRDX* amplicon was cloned into destination vector pK7WG2 by the Gateway cloning technique (Invitrogen) similarly to as described above.

### Determination of CabZIP63 subcellular localization

*Agrobacterium tumefaciens* strain GV3101 containing the constructs *35S::CabZIP63-GFP* and *35S::GFP* (used as a control) were grown overnight, and then resuspended in induction medium (10mM MES, 10mM MgCl_2_, pH 5.7, and 150 µM acetosyringone). Bacterial suspensions (OD_600_=0.8) were injected into *N. benthamiana* leaves using a syringe without a needle. At 48h post-infiltration (hpi), green fluorescent protein (GFP) fluorescence was imaged using a laser scanning confocal microscope (TCS SP8, Leica, Solms, Germany) with an excitation wavelength of 488nm and a 505–530nm band-pass emission filter.

### Transient overexpression of *CabZIP63* (*–SRDX*) or *CaWKRY40* (*–SRDX*) in pepper leaves

For transient overexpression analysis, *A. tumefaciens* strain GV3101 harboring the *35S::CabZIP63* (*–SRDX*) or *35S::CaWRKKY40* (–*SRDX*) vector was grown overnight, and then resuspended in induction medium. The bacterial suspension (OD_600_=0.8) was injected into leaves of pepper plants at the eight-leaf stage, and the injected leaves were harvested at 24 hpi for further use.

### VIGS of *CabZIP63* in pepper plants

For *CabZIP63* silencing analysis, the 3′-UTR of *CabZIP63* was used for VIGS vector construction; its sequence specificity was confirmed by genome-wide homology sequence searching by BLAST against sequences in the CM334 and Zunla-1 databases (http://peppergenome.snu.ac.kr/ and http://peppersequence.genomics.cn/page/species/blast.jsp). We did not find any homologous sequence in other pepper genes. The resulting *Tobacco rattle virus* (TRV)-based vectors TRV2-*CabZIP63* and TRV1 were transformed into *A. tumefaciens* strain GV3101. GV3101 cells harboring TRV1 and TRV2-*CabZIP63* or TRV2 as a negative control (resuspended in the induction medium at a 1:1 ratio, OD_600_=0.6) were co-infiltrated into cotyledons of 2-week-old pepper plants. The details of the process were as described in our previous studies (Dang *et al.*, 2014; Cai *et al.*, 2015; Zhang *et al.*, 2015b).

### Histochemical staining

Staining with trypan blue and DAB was carried out according to the previously published method of Choi *et al.* (2012), following the process as detailed in our previous studies (Dang *et al.*, 2014; Cai *et al.*, 2015; Liu *et al.*, 2015).

### Quantitative real-time RT–PCR

To determine the relative transcription levels of selected genes, real-time reverse transcription–PCR (RT–PCR) was performed with specific primers (Supplementary Table S3) according to the manufacturer’s instructions for the BIO-RAD Real-time RT-PCR system (Foster City, CA, USA) and the SYBR Premix Ex Taq II system (TaKaRa). Total RNA preparation and real-time RT–PCR were carried out following procedures used in our previous studies (Dang *et al.*, 2014; Cai *et al.*, 2015; Zhang *et al.*, 2015b). At least three replications of each experiment were performed. Data were analyzed by the Livak method (Livak and Schmittgen, 2001) and expressed as a normalized relative expression level (2^-ΔΔCT^) of the respective genes. The relative transcript levels of the analyzed pepper were normalized to the transcript levels of *CaACTIN* (GQ339766) and 18S rRNA (EF564281). In each case, three technical replications were performed for each of at least three independent biological replicates.

### Chromatin immunoprecipitation analysis

ChIP assays were performed as described by Cai *et al.* (2015). The GV3101 strain containing *35S::CabZIP63-HA* or *35S::CaWRKY40-HA* was infiltrated into the leaves of pepper plants at the eight-leaf stage; the plants were harvested and ~2g of pepper leaves were treated with either 10mM β-mercaptoethanol or DMSO (solvent control) for 16h and subsequently fixed with 1.0% formaldehyde for 5min. The chromatin was sheared to an average length of 500bp by sonication, and immunoprecipitated with antibody against hemagglutinin (HA; Santa Cruz Biotechnology). A 10mg aliquot of antibodies was used for each ChIP analysis, and the immunoprecipitated DNA was analyzed for enrichment of CabZIP63 or CaWRKY40 at the promoter region of target genes by quantitative real-time RT–PCR. Fold increases of immunoprecipitated DNA were calculated relative to the input DNA and the internal control *CaACTIN* or 18S rRNA. Each sample was quantified at least in triplicate. The primers used for real-time RT–PCR analysis in ChIP assays are listed in Supplementary Table S4.

### Fluorometric GUS enzymatic assay

A fluorometric GUS enzymatic assay for measuring GUS activity in pepper plant extracts was performed as described previously (Jefferson *et al.*, 1987). Leaves were lysed in extraction buffer (50mM phosphate buffer, pH 7.0, 10mM EDTA, 0.1% Triton X-100, 0.1% sodium lauryl sarcosine, and 10mM β-mercaptoethanol) by freezing with liquid nitrogen, and were ground using a pestle and mortar. Aliquots of the extracts (100 μl) were added to 1ml of assay buffer (extraction buffer containing 1mM MU), pre-warmed, and incubated at 37 °C. After 0, 5, and 20min of incubation, 100 μl samples were removed and placed in 1.9ml of stop buffer (200 μM sodium carbonate). Fluorescence was measured using a Multi Detection Microplate Reader (Bio-TEK Synergy™ HT, Bad Friedrichshall, Germany). The total protein content in plant extracts was estimated by the Bradford method using BSA as a standard (Bradford, 1976).

## Results

### Cloning and sequence analysis of CabZIP63

To isolate the possible TFs that transcriptionally modify the expression of CaWRKY40, a yeast one-hybrid system screening of a pepper cDNA library constructed from leaves of Zunla-1 using an ~400bp promoter region of *CaWRKY40* containing a G-box and a C-box as bait was performed. Among the 12 positive clones acquired, one clone turned out to be full-length cDNA of a gene encoding a bZIP protein. It is 1389bp in length, contains a 1272bp ORF, and a conserved bZIP domain was found in its deduced amino acid sequence and a nuclear localization signal (NLS) in its C-terminus. It shared 62.56, 56.74, 53.86, 52.74, 52.74, 51.88, and 52.93% deduced amino acid sequence identities with the products of *NtbZIP1*, *CsbZIP6*, *PtbZIP*, *GmSBZ1*, *GsCPRF2*, *GaCPRF2*, and *MtbZIP88*, respectively. Since it exhibited the highest sequence identity (43%) with that of the product of *AtbZIP63* among all of the bZIPs in Arabidopsis, it was designated *CabZIP63* (*Capsicum annuum bZIP63*) (see Supplementary Fig. S1). Although *AtbZIP63* was found to act as a sensitive integrator of transient ABA and glucose signals (Matiolli *et al.*, 2011), the role of *CabZIP63* in pepper has not been characterized so far.

### CabZIP63 is localized to nuclei

An NLS (_397_LEHLQKRIRGD_407_, Supplementary Fig. S1) present in CabZIP63 indicates its nuclear localization; to confirm this probability, we assayed its subcellular localization by expression of the constructs *p35S::CabZIP63-GFP* and *p35S::GFP* (control) individually in *N. benthamiana* leaves. Typical results showed the exclusive localization of CabZIP63–GFP in nuclei, whereas the GFP control was observed in multiple subcellular compartments including the cytoplasm and nuclei (Supplementary Fig. S2).

### The expression of *CabZIP63* was enhanced by HTHH and RSI as well as exogenous applied SA, MeJA, ETH, and ABA

Since CaWRKY40 acts as positive regulator in pepper’s response to HTHH and RSI, and as CabZIP63 can bind to the *CaWRKY40* promoter, CabZIP63 is likely to play a role in the above defense responses. To test if this is the case, the activity of the *CabZIP63* promoter in response to HTHH and RSI was examined using *Agrobacterium*-mediated transient overexpression in pepper leaves. The construct *pCabZIP63::GUS* was transformed into *Agrobacterium* GV3101, and cells containing *pCabZIP63::GUS* were infiltrated into pepper leaves, at 24 hpi. The infiltrated pepper leaves were further inoculated with *R. solanacearum* (OD_600_=0.6) or treated with heat stress (38 °C) under 90% humidity; after RSI or HTHH treatment, the leaves were harvested at appropriate time points and the GUS activities were measured in the pepper leaves. The results showed that both RSI and HTHH significantly up-regulated the expression of *CabZIP63* compared with the mock treatment (Supplementary Fig. S3A). The relative transcription levels of *CabZIP63* were also measured at appropriate time points after RSI or HTHH by real-time RT–PCR, and the result showed that the transcript levels of *CabZIP63* were enhanced more intensively than those of *pCabZIP63*-driven GUS by RSI or HTHH; this might be due to post-transcriptional regulation of GUS expression and the difference in feedback regulation between expression of GUS and CabZIP63 *in planta* (Supplementary Fig. S3B). As phytohormones such as SA, JA, ET, and ABA have typically been found to be involved in plant defense signaling against different biotic and abiotic stresses, to test if *CabZIP63* is involved in the pathways mediated by these hormones, the expression of *pCabZIP63*-driven GUS after exogenous applications of SA, MeJA, ETH, and ABA was measured. The results showed that GUS expression was induced by all of the four test hormones (Supplementary Fig. S3C).

### *CabZIP63* is transcriptionally regulated by CabZIP63 itself

**Fig. 1.**
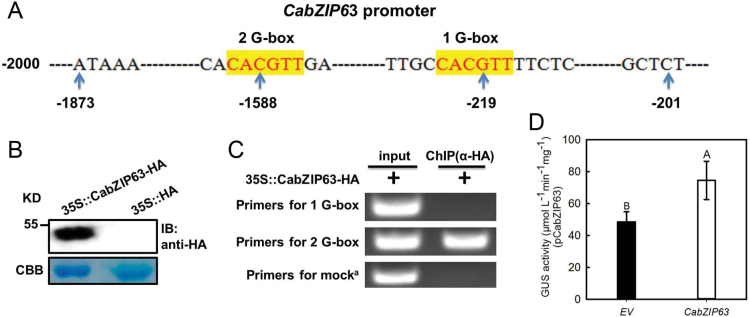
*CabZIP63* is transcriptionally regulated by CabZIP63 itself. (A) Schematic representation and sequence of elements within the –200 to –1873bp region of *pCabZIP63*. (B) Transient overexpression of *CabZIP63-HA* in pepper leaves as detected by immunoblotting (IB). CBB, Coomassie Brilliant Blue. (C) ChIP assay indicated that CabZIP63 binds to its own promoter. Pepper leaves were infiltrated with GV3101 cells carrying *35S::CabZIP63-HA*. The infiltrated leaves were harvested and cross-linked with 1% formaldehyde for chromatin preparation. The sheared chromatin was immunoprecipitated with an anti-HA antibody. The acquired DNA samples were adjusted to the same concentration and PCRs were performed using specific primer pairs according to flanking sequences of the two G-boxes. Lanes 1, input (total DNA–protein complex); lanes 2, DNA–protein complex immunoprecipitated with an anti-HA antibody. Mock^a^ (Supplementary Table S4) is a DNA fragment that was distant from the *cis*-element of the two G-boxes in *pCabZIP63*, and was used as a control for ChIP assay. (D) The expression of *pCabZIP63*-driven GUS was induced by transient overexpression of *CabZIP63* in pepper leaves. Data represent the means ±SD from four independent biological replicates. Different upper case letters indicate significantly different means, as analyzed by Fisher’s protected LSD test (*P*<0.01). (This figure is available in colour at *JXB* online.)

The presence of a G-box and C-box, which were previously found to be bound by bZIP TFs (Izawa *et al.*, 1993), in the promoter region of *CabZIP63* implies that the transcriptional expression of *CabZIP63* is probably self-regulated. To test this possibility, we performed a ChIP assay to test if *CabZIP63* can also bind its own promoter. GV3101 cells containing *35S::CabZIP63-HA* were infiltrated into the leaves of pepper plants; 48h later, the leaves were harvested for chromatin preparation and ChIP analysis. The stable expression of fused protein CabZIP63-HA was confirmed by western blot analysis with anti-HA antibody. The result showed that a specific primer pair flanking one of the two G-boxes amplified the product of the DNA fragments immunoprecipitated by anti-HA antibody as templates, indicating that CabZIP63 can bind to it own promoter (Fig. 1A–C). To test the possible self-regulation of *CabZIP63* further, *pCabZIP63*-driven GUS expression was analyzed after transient overexpression of *CabZIP63* in pepper plants, and the result showed that a significantly enhanced expression of GUS was triggered by transient overexpression of *CabZIP63* (Fig. 1D).

### Effect of *CabZIP63* silencing on resistance of pepper to *R. solanacearum* and thermotolerance, and the expression of marker genes

**Fig. 2.**
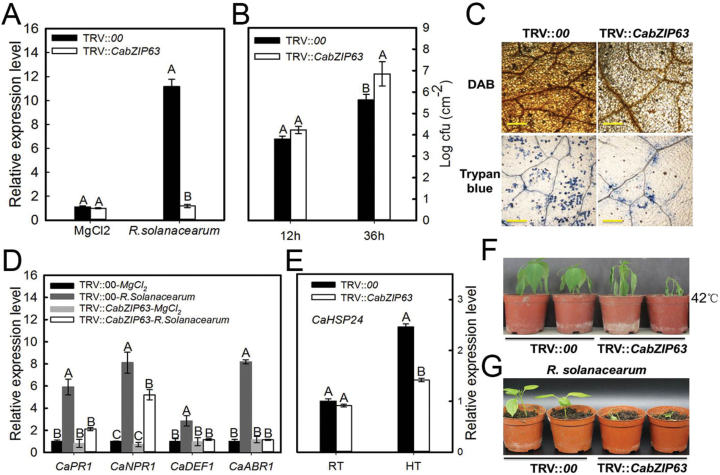
Distinct responses of *CabZIP63*-silenced pepper plants to RSI and HTHH. (A) Real-time RT–PCR analysis of *CabZIP63* expression in *R. solanacearum*-inoculated or mock-treated (inoculated with solution of MgCl_2_) *CabZIP63*-silenced pepper (TRV::*CabZIP63*) and control (TRV::*00*). (B) Detection of growth of *R. solanacearum* in *CabZIP63*-silenced or control pepper plants inoculated with *R. solanacearum* at 12h and 36h. (C) Trypan blue staining and DAB staining in *R. solanacearum*-inoculated *CabZIP63*-silenced (TRV::*CabZIP63*) and empty vector (TRV::*00*) pepper leaves at 2 days post-inoculation (dpi). Scale bars=50 μm. (D) Real-time RT–PCR analyses of transcription levels of the tested defense-related genes in *CabZIP63*-silenced pepper (TRV::*CabZIP63*) and control (TRV::*00*) after inoculation with or without *R. solanacearum*. (E) Real-time RT–PCR analyses of transcription levels of the thermotolerance-related *CaHSP24* in *CabZIP63*-silenced pepper (TRV::*CabZIP63*) and control (TRV::*00*) with or without high temperature (HT) treatment. (F) The pepper plants were treated at 42 °C for 24h, and then kept under normal temperature conditions (25 °C) for 24h before checking the phenotype. (G) Phenotypic effect of *R. solanacearum* attack on *CabZIP63*-silenced (TRV::*CabZIP63*) and control (TRV::*00*) plants at 14 dpi. Data represent the means ±SD from four independent experiments. Different letters indicate significant differences, as determined by Fisher’s protected LSD test (*P*<0.01). (This figure is available in colour at *JXB* online.)

To test the role of *CabZIP63* in immunity and thermotolerance under high humidity, we performed loss-of-function experiments in pepper seedlings in which *CabZIP63* was silenced by VIGS. We used TRV::*CaPDS*, which silences the phytoene desaturase (PDS) gene and induces a photobleaching phenotype, as an additional control to determine the success of gene silencing. The two vectors TRV1 (PYL192) and TRV2 (PYL279) were separately transformed into *A. tumefaciens* GV3101. The two resulting GV3101 strains were mixed and co-injected into leaves of pepper seedlings, and seedlings were incubated at 16 °C for 56h; after this they were kept at 25 °C. Four independent experiments were performed, and we obtained ~100 plants of TRV::*00* and 100 plants of TRV::*CabZIP63*, respectively. Six plants were randomly selected to check the efficiency of gene silencing by inoculation with cells of the virulent *R. solanacearum* strain FJC100301, which was detected by root irrigation to be virulent to pepper plants using two pepper inbred lines, GZ03, a line moderately resistant to *R. solanacearum*, and XJ116, a line susceptible to *R. solanacearum* (Supplementary Fig. S4). The result showed that in FJC100301-challenged TRV::*CabZIP63* pepper plants, *CabZIP63* transcript levels were reduced to ~30% of those in TRV::*00* plants, suggesting the success of *CabZIP63* silencing (Fig. 2A). With these *CabZIP63*-silenced pepper plants, the effects of *CabZIP63* silencing on pepper immunity and thermotolerance were assayed; 60 TRV2::*CabZIP63* and 60 TRV2::*00* plants were randomly selected and inoculated with FJC100301. Definite wilting symptoms were observed in TRV2::*CabZIP63* plants at 14 dpi, with an average disease index of 3.0, while TRV2::*00* plants exhibited only faint wilting symptoms, with an average disease index of 1.2 (Supplementary Table S5). Consistently, our data also showed that the growth of *R. solanacearum* was significantly increased in *CabZIP63*-silenced pepper plants, manifested by higher cfu values compared with those in the control plants at 36 hpi (Fig. 2B), and dark-brown DAB (indicator of H_2_O_2_ accumulation) and trypan blue (indicator of cell death or necrosis) staining was detected in the leaves of TRV2:*00* plants at 48 hpi, whereas the intensities of DAB and trypan blue staining were distinctly reduced in *CabZIP63*-silenced leaves (Fig. 2C). Additionally, the expression of defense-related *CaPR1*, *CaNPR1*, *CaDEF1*, and *CaABR1* was significantly lower in leaves of *R. solanacearum*-inoculated *CabZIP63*-silenced pepper plants at 24 hpi compared with that in control plants (Fig. 2D). On the other hand, when challenged with high temperature (42 °C) under 90% humidity, the (i) TRV2::*CabZIP63* plants exhibited significantly increased thermosensitivity compared with the wild-type control plants; (ii) the thermotolerance-associated *CaHSP24* was much lower in TRV2::*CabZIP63* plants compared with the TRV2::*00* plants (Fig. 2E). The results strongly suggest that silencing of *CabZIP63* significantly impairs resistance/tolerance of pepper plants to RSI or HTHH (Fig. 2F, G).

### Transient overexpression of *CabZIP63* or *CabZIP63–SRDX* modifies the cell death, immunity and thermotolerance-associated marker gene expression in pepper plants

**Fig. 3.**
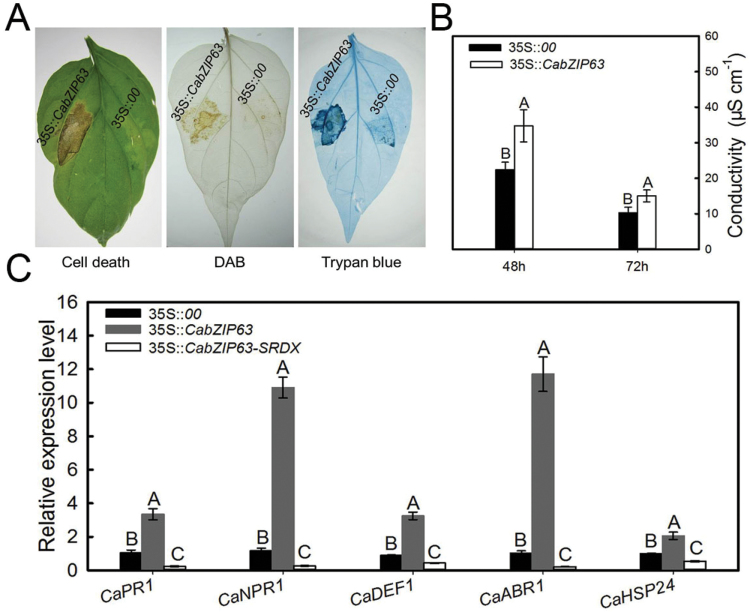
Cell death and expression of immunity- or thermotolerance-related marker genes were triggered by transient overexpression of *35S::CabZIP63*. (A) Cell death triggered by transient overexpression of *35S::CabZIP63*, displayed with phenotype, DAB staining, and trypan blue staining at 4 dpi, respectively. (B) Quantification of electrolyte leakage as ion conductivity to assess the cell death response in leaf discs. (C) Quantitative real-time RT–PCR analysis of the expression of immunity- or thermotolerance associated marker genes in *35S::CabZIP63* and *35S::CabZIP63-SRDX* expressed in pepper leaves at 24 hpi, respectively. Data represent the means ±SD from four independent biological replicates. Different letters above the bars indicate significantly different means (*P*<0.01), as analyzed by Fisher’s protected LSD test. (This figure is available in colour at *JXB* online.)

To confirm further the finding that *CabZIP63* acts as a positive regulator in pepper’s defense response to both HTHH and RSI, *CabZIP63* was transiently expressed in pepper leaves by infiltration with GV3101 cells carrying 35S::*00* (empty vector) or 35S::*CabZIP63*. Real-time RT–PCR and immunoblot analysis showed that the HA-tagged CabZIP63 mRNA and protein were stably expressed in pepper plants (Fig. 5C). HR-mediated cell death and H_2_O_2_ accumulation were assessed by staining with trypan blue and DAB, respectively; the result showed that the transient overexpression of *CabZIP63* induced both extensive HR-mediated cell death and accumulation of H_2_O_2_ in pepper plants (Fig. 3A). We also detected ion leakage to measure the severity of cell necrosis caused by transient overexpression of *CabZIP63*, and the result showed that pepper leaves transiently overexpressing *CabZIP63* exhibited more ion leakage at 48 and 72 hpi than leaves expressing the empty vector control (Fig. 3B). We also examined changes in the expression of defense-related genes including *CaNPR1*, *CaPR1*, *CaDEF1*, and *CaHSP24*, and the results showed that the relative transcription levels of *CaPR1*, *CaNPR1*, *CaDEF1*, and *CaHSP24* increased continuously during transient overexpression of *CabZIP63*. In contrast, the transient overexpression of *CabZIP63*-*SRDX*, a repressor version of *CabZIP63*, was also performed in pepper leaves, and the success of fused *CabZIP63-SRDX* mRNA was confirmed by real-time RT–PCR. The result showed that the overexpression of *CabZIP63-SRDX* markedly decreased the expression of the tested marker genes (Fig. 3C). These data indicated that CabZIP63 might act as a positive regulator in the response of pepper to pathogen and heat stress.

### CabZIP63 binds to both the G-box and the C-box in the promoter of *CaWRKY40*

**Fig. 4.**
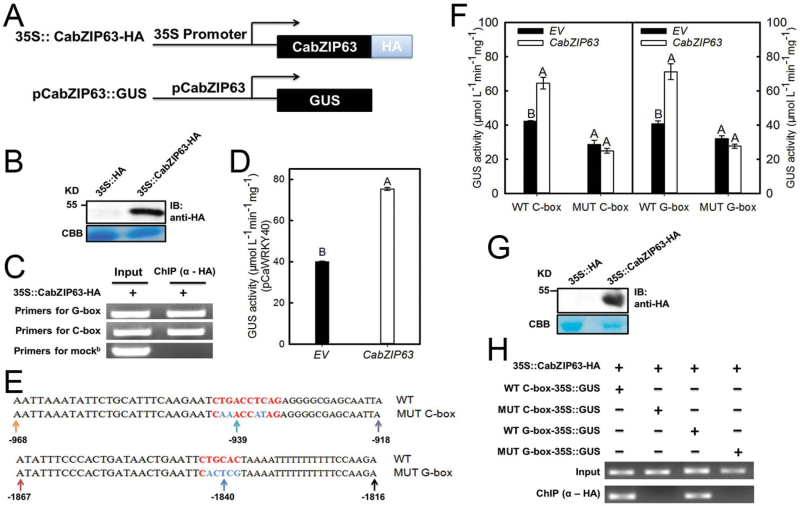
The binding of CabZIP63 to *pCaWRKY40* and GUS expression in a C- or G-box-dependent manner. (A) Schematic diagram of transient overexpression and GUS reporter constructs used for co-transfection in pepper leaves. (B) Transient overexpression of *CabZIP63-HA* in pepper leaves for ChIP assay as detected by immunoblotting (IB). CBB, Coomassie Brillaint Blue. (C) ChIP assay indicated that CabZIP63 bound to *pCaWRKY40*. Chromatin was isolated from infiltrated pepper leaves cross-linked with 1% formaldehyde, and was sheared, and immunoprecipitated with an anti-HA antibody. The acquired DNA samples adjusted to the same concentration were used as templates for PCRs with specific primer pairs based on C-box- or G-box-flanking sequences. Lanes 1, input (total DNA–protein complex); lanes 2, DNA–protein complex immunoprecipitated with an anti-HA antibody. Mock^b^ (Supplementary Table S4) is a DNA fragment distant from the C-box and G-box in *pCaWRKY40*, and was used as a control for ChIP assay. (D) *pCaWRKY40*-driven GUS expression was triggered by transient overexpression of *CabZIP63* by *Agrobacterium* infiltration in pepper leaves. The pepper leaves were co-infiltrated with GV3101 cells carrying *pCaWRKY40::GUS* or *35S::CabZIP63*. (E) Schematic representation and sequence of elements that were mutagenized within the –918 to –968bp and –1816 to –1867bp regions in *pCaWRKY40*; the fragment harboring each C- or G-box and their corresponding mutants were amplified by PCR and were cloned into vector pMDC163, in which the original CaMV35S promoter was replaced by the core promoter of CaMV35S (–46bp to +8bp). The acquired vectors were named pW40C, pW40CM, pW40G, and pW40GM, respectively. (F) The constructs of pW40C, pW40CM, pW40G, and pW40GM were transformed into GV3101 individually, and the resulting cells containing the individual constructs were co-infiltrated with GV3101 cells containing *35S::CabZIP63-HA* into pepper leaves, which were harvested for GUS expression assay at the appropriate time points. (G) Transient overexpression of *CabZIP63-HA* in *N. benthamiana* leaves for ChIP assay as detected by immunoblotting (IB). (H) The result of ChIP indicated that CabZIP63 failed to bind to the mutant C- or G-box. GV3101 cells containing *35S::CabZIP63-HA* were co-infiltrated into *N. benthamiana* leaves with GV3101 cells carrying pW40C, pW40CM, pW40G, and pW40GM for ChIP assay. Data represent the means ±SD from four independent biological replicates. Different upper case letters indicate significantly different means, as analyzed by Fisher’s protected LSD test (*P*<0.01). (This figure is available in colour at *JXB* online.)

As CabZIP63 responded transcriptionally to HTHH and RSI similarly to CaWRKY40 (Dang *et al.*, 2013), and the G-box and C-box, which were previously found to be preferentially bound by bZIPs (Izawa *et al.*, 1993), are present in the promoter region of *CaWRKY40*, we speculated that *CaWRKY40* might be transcriptionally regulated by CabZIP63 in pepper’s response to HTHH and RSI. To test this possibility, we performed ChIP analysis to test if CabZIP63 can also bind the promoter of *CaWRKY40* via the G-box or C-box. The result showed that the fused CabZIP63-HA was successfully expressed by transient overexpression in pepper plants, and both the specific primers pairs flanking the G-box or the C-box amplified products with the DNA fragments immunoprecipitated by anti-HA antibody as templates, indicating that CabZIP63 can bind to the *CaWRKY40* promoter (Fig. 4A–D). To confirm this result further and to determine whether the C-box or the G-box or both the boxes are responsible for the specific binding, we constructed pW40C, pW40G, pW40CM, and pW40GM, and analyzed the possible contribution of binding of CabZIP63 to the C-box or G-box in the transcriptional regulation of *CaWRKY40* by CabZIP63. The results showed that the overexpression of *CabZIP63* activated the expression of GUS driven by pW40C and pW40G, but failed to activate the expression of GUS driven by either pW40CM or pW40GM (Fig. 4E, F). With these four vectors, we also tested the possible binding of CabZIP63 to the C- or G-box by ChIP through transient overexpression in *N. benthamiana* leaves; the results showed that CabZIP63 bound to both the C-box and the G-box (Fig. 4G, H).

### The effect of RSI or HTHH on the binding of CabZIP63 to *pCaWRKY40*

As *CabZIP63* is enhanced by both HTHH and RSI, and CabZIP63 binds to the promoter of *CaWRKY40*, the binding of CabZIP63 to the promoter of *CaWRKY40* might contribute to the transcriptional activation of *CaWRKY40* against RSI or HTHH. To test this possibility, the binding of CabZIP63 to *pCaWRKY40* under RSI or HTHH was assayed by ChIP, during which *35S::CabZIP63-HA* was transiently overexpressed in pepper leaves by *Agrobacterium* infiltration. The infiltrated leaves were harvested at 48 hpi for chromatin preparation, excision, and immunoprecipitation, and the resulting DNA fragments were used as template for real-time RT–PCR with specific primer pairs for *pCaWRKY40* (Supplementary Fig. S5). The results showed that both RSI and HTHH enhanced the binding of CabZIP63 to *pCaWRKY40*.

### Inter-relationship between the expression of *CabZIP63* and *CaWRKY40* at the transcriptional level

**Fig. 5.**
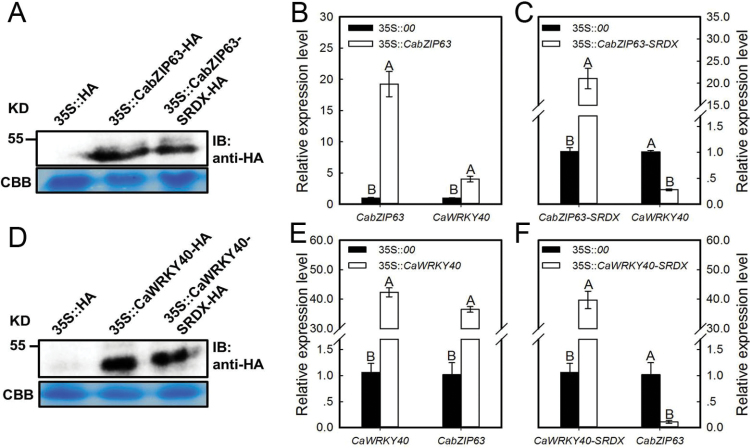
Inter-relationship between the expression of *CabZIP63* and *CaWRKY40* at the transcriptional level. (A) The effect of transient overexpression of *35S::CabZIP63* on the transcript level of *CaWRKY40* in pepper leaves. (B) The effect of transient overexpression of *35S::CabZIP63-SRDX* on the transcript level of *CaWRKY40* in pepper leaves. (C) Transient overexpression of CabZIP63-HA and CabZIP63-SRDX-HA in pepper leaves as detected by immunoblotting (IB). CBB, Coomassie Brilliant Blue. (D) The effect of transient overexpression of *35S::CaWRKY40* on the transcript level of *CabZIP63* in pepper leaves. (E) The effect of transient overexpression of *35S::CaWRKY40-SRDX* on the transcript level of *CabZIP63* in pepper leaves. (F) Transient overexpression of *CaWRKY40-HA* and *CaWRKY40-SRDX-HA* in pepper leaves as detected by immunoblotting. The pepper leaves were infiltrated with GV3101 cells (OD_600_=0.8) containing different constructs, which were harvested at 24 hpi for total RNA extraction; the transcript levels of *CaWRKY40* or *CabZIP63* were determined by real-time RT–PCR with specific primer pairs. Data represent the means ±SD from four independent biological replicates. Different upper case letters indicate significantly different means, as analyzed by Fisher’s protected LSD test (*P*<0.01). (This figure is available in colour at *JXB* online.)

As both *CabZIP63* and *CaWRKY40* are up-regulated transcriptionally by RSI and HTHH, and CabZIP63 binds to *pCaWRKY40*, it is presumed that *CabZIP63* might be transcriptionally up-regulated by both RSI and HTHH and could then activate the transcription of *CaWRKY40*. To test this possibility, the effect of transient overexpression of *CabZIP63* in pepper leaves on the transcript level of *CaWRKY40* was measured by real-time RT–PCR, and the results showed that the transient overexpression of *CabZIP63* enhanced the transcript level of *CaWRKY40* in pepper leaves (Fig. 5A, B). In contrast, transient overexpression of *CabZIP63-SRDX* was performed in pepper plants, and the transcript and protein of *CabZIP63-SRDX* were confirmed by real-time RT–PCR and western blot analysis, respectively. The result showed that the overexpression of *CabZIP63-SRDX* significantly decreased the transcription level of *CaWRKY40* by real-time RT–PCR using their corresponding specific primers designed according to the sequence in its 3′-UTR (Fig. 5A, C). Consistently, *pCaWRKY40*-driven GUS expression assay showed that the GUS expression was significantly promoted by transient overexpression of *CabZIP63* (Fig. 4D). Interestingly, our data also showed that the transient overexpression of *CaWRKY40* enhanced the transcription level of *CabZIP63* in pepper leaves (Fig. 5D, E). In contrast, the transient overexpression of *CaWRKY40-SRDX*, which was confirmed by both real-time RT–PCR and western blot analysis, significantly decreased the transcription level of endogenous *CaWRKY40* and *CabZIP63* by real-time RT–PCR using the specific primers designed according to of the sequence in their 3′-UTRs (Fig. 5D, F; Supplementary Fig. S6A, B).

### The effect of transient overexpression of *CabZIP63* on the binding of CaWRKY40 to the promoters of its target genes

**Fig. 6.**
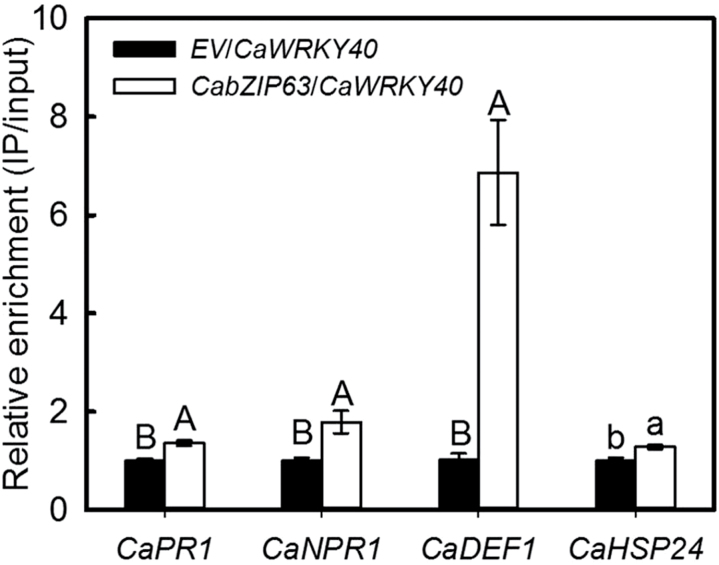
The DNA binding of CaWRKY40 to the promoters of its target genes was potentiated by transient overexpression of *35S::CabZIP63* in pepper plants. GV3101 cells containing *35S::CaWRKY40-HA* and *35S::CabZIP63-83* were mixed at a ratio of 1:1 and were co-infiltrated into pepper leaves, with GV3101 cells containing 35S::*00* as mock treatment. The leaves were harvested at 48 hpi for chromatin preparation (relative enrichment levels of samples of CaWRKY40 were set to 1 after normalization by input). Data represent the means ±SD from three independent biological replicates. Different upper case letters indicate significant differences from three independent biological replicates based on the LSD test (*P*<0.01). Different lower case letters indicate significant differences from three independent experiments based on the LSD test (*P*<0.05).

Our previous data showed that *CaWRKY40* achieves its function in pepper’s response to RSI and HTHH by transcriptional modification of its targets genes including *CaPR1*, *CaNPR1*, *CaDEF1*, and *CaHSP24*, and data in the present study showed that *CabZIP63* manipulates the expression of *CaWRKY40*, implying that the up-regulation of *CabZIP63* might ultimately enhance the binding of CaWRKY40 to the promoters of its target genes. To confirm this possibility, we assayed the effect of *CabZIP63* overexpression on the direct binding of CaWRKY40 to the promoters of its target genes by transient overexpression in pepper leaves. The results showed that the enrichment of CaWRKY40 at the promoters of *CaNPR1*, *CaPR1*, *CaDEF1*, and *CaHSP24* was significantly enhanced by transient overexpression of *CabZIP63* compared with the control pepper leaves (Fig. 6).

## Discussion

In our previous study, *CaWRKY40* was found to be up-regulated transcriptionally by HTHH or RSI, and to act as a positive regulator in pepper’s response to these stresses (Dang *et al.*, 2013); however, their underlying mechanism remained to be elucidated. In the present study, we provide evidence that *CabZIP63*, a member of the bZIP family in pepper, acts as a positive TF modulating the expression of *CaWRKY40*, forming a positive feedback loop with *CaWRKY40* during pepper’s defense response to HTHH or RSI.

The evidence that CabZIP63 is a bZIP family member comes from the following. First, CabZIP63 contains a conserved domain, which is generally present in bZIP proteins, in its deduced amino acid sequence, and also exhibits sequence identity with bZIP orthologs in other plant species such as, for example, bZIP63 in Arabidopsis, bZIP1 in *Nicotiana tabacum*, bZIP6 in *Camellia sinensis*, SBZ1 in *Glycine max*, and bZIP88 in *Medicago truncatula*. Secondly, CabZIP63 contains an NLS in its C-terminus and was consistently localized in the nuclei in cells of *N. benthamiana* leaves during subcellular localization using transient overexpression by *Agrobacterium* infiltration, which is a general characteristic of the majority of TFs (Boulikas, 1994) including bZIPs (Liu *et al.*, 2012). Thirdly, CabZIP63 was found to bind to the G-box- and C-box-containing *CaWRKY40* promoter by ChIP in the present study, and can activate the expression of a GUS reporter gene in a G-box- or C-box-dependent manner. In a previous study, bZIPs were found to bind preferentially via their basic region to DNA sequences with an ACGT core *cis*-element, in particular like the G-box, C-box, and A-box (Izawa *et al.*, 1993; Foster *et al.*, 1994; Heinekamp *et al.*, 2002). All these results indicate that *CabZIP63* is a member of the pepper bZIP family.

Accumulated evidence indicates that plant growth, development, and response to environmental stress are largely regulated at the transcriptional level (Baena-Gonzalez *et al.*, 2007; Baena-Gonzalez and Sheen, 2008; Rymen and Sugimoto, 2012; Buscaill and Rivas, 2014), and genes up-regulated during plant response to stresses can have important roles in plant resistance/tolerance to stresses (Bartsch *et al.*, 2006; Dang *et al.*, 2013, 2014; Cai *et al.*, 2015; Sun *et al.*, 2015). Our data showed that the expression of the GUS reporter gene driven by the *CabZIP63* promoter was induced by RSI or HTHH. Consistently, the transcript level of *CabZIP63* was also found to be enhanced by both RSI and HTHH, implying that CabZIP63 might act as a positive regulator in pepper’s defense response to these stresses. This possibility was corroborated by the data from the loss- and gain-of function study of *CabZIP63*. Pepper plants with *CabZIP63* silenced by VIGS exhibited a significantly decreased resistance and tolerance to RSI and HTHH compared with control plants, accompanied by down-regulation of thermotolerance-associated *CaHSP24* (Pivovarova *et al.*, 2005; Baek *et al.*, 2014) under HTHH, and down-regulation of immunity-associated *CaNPR1* (Ustun *et al.*, 2013), *CaPR1* (Kim and Hwang, 2012), *CaDEF1* (Choi *et al.*, 2008), and *CaABR1* (Choi and Hwang, 2011) under RSI. In contrast, the transient overexpression of *CabZIP63* activated HR-mediated cell death compared with the control, revealed by a high level of ion leakage and darker trypan blue and DAB staining (Choi *et al.*, 2012; Dang *et al.*, 2013), coupled with up-regulation of *CaABR1*, *CaPR1*, *CaNPR1*, *CaDEF1*, and *CaHSP24* in pepper leaves transiently overexpressing *CabZIP63*. These results strongly suggest that CabZIP63 acts as positive regulator in pepper’s response to both HTHH and RSI. Although bZIPs have been extensively studied in plant immunity (Pontier *et al.*, 2001; Kim and Delaney, 2002; Zander *et al.*, 2010; Du *et al.*, 2014), and in abiotic stresses such as salinity (Orellana *et al.*, 2010; Lakra *et al.*, 2015; Zhang *et al.*, 2015a), drought (Orellana *et al.*, 2010; Lakra *et al.*, 2015), cold (Hwang *et al.*, 2005; Zhang *et al.*, 2015a), and heat (Li *et al.*, 2012; Srivastava *et al.*, 2014), and AtbZIP63 was previously found to be a sensitive integrator of transient ABA and glucose signals, as well as low energy response (Matiolli *et al.*, 2011; Mair *et al.*, 2015), no information is available about the involvement of bZIPs in responses of plants to both pathogen infection and heat stress.

Our data also indicate a close relationship between *CabZIP63* and *CaWRKY40*, as they showed a synergistic response not only to RSI and HTHH, but also to exogenously applied SA, MeJA, ETH, and ABA (Dang *et al.*, 2013). Importantly, CabZIP63 was found to bind *pCaWRKY40* and activated GUS expression in a C- and G-box-dependent manner, and this binding was found to be potentiated by both RSI and HTHH, suggesting that CabZIP63 acts as a TF of *CaWRKY40*. Since a multitude of *cis*-elements including a W-, G-, and C-box are present in *pCaWRKY40*, and CaWRKY6 was previously found to act as a positive regulator by directly activating the transcriptional expression of *CaWRKY40* during pepper response to RSI and HTHH (Cai *et al.*, 2015), indicating that the actions of *CaWRKY40* are orchestrated by multiple TFs, how these TFs co-ordinate to fine-tune the expression of *CaWRKY40* remains to be determined. In the present study, positive feedback loops were found in the transcriptional regulation of *CabZIP63* and between *CabZIP63* and *CaWRKY40*. The expression of *CabZIP63* was found to be induced by the transient overexpression of *CabZIP63* itself and by that of *CaWRKY40*, while it was decreased by the transient overexpression of *CabZIP63-SRDX* or *CaWRKY40-SRDX*, in which the *SRDX* domain was used to transform CabZIP63 and CaWRKY40 into dominant-negative repressor versions and has been widely used to assess the roles of TFs (Grunewald *et al.*, 2013; Takada, 2013; Figueroa and Browse, 2015). This suggests an indirect regulation of *CabZIP63* by CaWRKY40 possibly via unknown upstream components, since CaWRKY40 failed to bind to *pCabZIP63* (data not shown). Similar positive feedback loops have also been found during pepper’s response to RSI and HTHH between *CaWRKY6* and *CaWRKY40* (Cai *et al.*, 2015), as well as in plant response to a wide array of stresses (Miersch and Wasternack, 2000; Arimura *et al.*, 2002; Yan *et al.*, 2015; Zhang *et al.*, 2015b). For example, positive feedback loops have been found in signaling against stresses mediated by brassinosteroid (BR) (Yan *et al.*, 2015) and ABA (Xiong *et al.*, 2002), as well as JA and ET (Arimura *et al.*, 2002). These positive feedback loops might allow plants to respond to stresses more efficiently. Interestingly, compared with up-regulation of *CabZIP63* by the transient overexpression of *CaWRKY40*, the transient overexpression of *CabZIP63* only slightly activated the transcriptional expression of *CaWRKY40*, as the function of bZIPs has been found to be modified by other proteins such as NPR1, WRKY TFs, and other bZIPs in a protein–protein interaction manner (Spoel *et al.*, 2003; Alves *et al.*, 2013; Caarls *et al.*, 2015). We speculate that the lower level of *CaWRKY40* activation by transient overexpression of *CabZIP63* might be due to the absence of its interacting partners under this condition. In addition to the transcription level, *CaWRKY40* appears to be modulated by *CabZIP63* at the post-transcriptional level, since the binding of CaWRKY40 to its target genes was found to be up-regulated by transient overexpression of *CabZIP63* in pepper leaves in the present study; however, the underlying mechanism remains to be elucidated. The above results also collectively suggest a close link between RSI resistance and HTHH tolerance in pepper plants, which might occur in multiple nodes including *CaWRKY6*, *CaWRKY40*, and *CabZIP63*, as well as other unidentified components, and these components may be functionally connected, forming a transcriptional network composed of positive and negative feedback loops and feed-forward modules. This arrangement may provide great regulatory potential for plants to trigger appropriate disease resistance and thermotolerance, and co-ordinate different biological processes including growth, development, and response to other stresses. The synergistic response of pepper to HTHH and pathogen infection might be a result of its evolution under simultaneously or alternately occurring HTHH and pathogen infection in their natural habitats.

Our data also showed that *CaNPR1* is a target of CaWRKY40, the expression of *CabZIP63* and the binding of CabZIP63 to *pCaWRKY40* was up-regulated by HTHH and RSI, and expression of *CaWRKY40* was also enhanced by transient overexpression of *CabZIP63*, suggesting that the up-regulation of *CabZIP63* by HTHH and RSI might result in accumulation of CaNPR1. As CaNPR1 acts as an important regulator by interacting with TGA, a member of the bZIP family, in plant immunity mediated by SA signaling (Spoel *et al.*, 2003; Caarls *et al.*, 2015), we speculate that CaNPR1 might interact with CabZIP63, which may play a role in modulating expression of *CaWRKY40*; further investigation is required to confirm this hypothesis and to elucidate the possible underlying mechanism.

Collectively, our data indicate that CabZIP63 acts as an activator of *CaWRKY40* in the response of pepper to RSI or HTHH. Upon exposure of RSI or HTHH, the expression of *CabZIP63* and its binding to *pCaWRKY40* are up-regulated; therefore, the expression of *CaWRKY40* and the binding of CaWRKY40 to its target genes are also activated, and eventually lead to the transcriptional modulation of target genes of CaWRKY40 and the defense response of pepper to RSI and HTHH. Our results will facilitate the dissection of the crosstalk between pepper’s response to HTHH and *R. solanacearum*.

## Supplementary data

Supplementary data are available at *JXB* online

Figure S1. Comparison of amino acid sequences deduced from pepper *CabZIP63* with the representative closely related proteins from other plant species.

Figure S2. The expression of *CabZIP63* was induced by RSI and HTHH as well as exogenously applied SA, MeJA, ETH, or ABA.

Figure S3. *CabZIP63* is transcriptionally regulated by CabZIP63 itself.

Figure S4. The detection of virulence of *R. solanacearum* strain FJC100301 by root irrigation.

Figure S5. The binding of CabZIP63 to *pCaWRKY40* and GUS expression in a C- or G-box-dependent manner.

Figure S6. Relative expression level of endogenous *CabZIP63* or *CaWRKY40* in pepper leaves transiently overexpressing *CabZIP63-SRDX* or *CaWRKY40-SRDX* by real-time RT–PCR.

Table S1. Grading standards for evaluation of disease resistance of pepper plants to *R. solanacearum* by root irrigation.

Table S2. Pepper primers used for vector construction in this study.

Table S3. Pepper primers used for real-time RT–PCR in this study.

Table S4. Pepper primers used for ChIP PCR or real-time RT–PCR in this study.

Table S5. The plant numbers with different levels of disease resistance in the VIGS assay of *CabZIP63* against *R. solanacearum* strain FJC100301 inoculation.

Supplementary Data

## Abbreviations:

dpi: days post-inoculation;
hpi: hours post-inoculation
HTHH: high temperature–high humidity
RSI: *Ralstonia solanacearum* inoculation
TRV: *Tobacco rattle virus*
VIGS: virus-induced gene silencing.
