# Supplementary material for: Pepper CabZIP63 acts as a positive regulator during Ralstonia solanacearum or high temperature–high humidity challenge in a positive feedback loop with CaWRKY40
Source: J Exp Bot. 2016 Mar 1;67(8):2439–51. doi: 10.1093/jxb/erw069 (PMC4809298; doi:10.1093/jxb/erw069)
Supplement: Supplementary Data [file supp_67_8_2439__index.html]

Pepper CabZIP63 acts as a positive regulator during Ralstonia solanacearum or high temperature–high humidity challenge in a positive feedback loop with CaWRKY40 — Pepper CabZIP63 acts as a positive regulator during Ralstonia solanacearum or high temperature–high humidity challenge in a positive feedback loop with CaWRKY40 — Supplementary Data 

# Pepper CabZIP63 acts as a positive regulator during *Ralstonia solanacearum* or high temperature–high humidity challenge in a positive feedback loop with CaWRKY40

## Supplementary Data

Data files

- supplementary\_figures\_S1\_S6\_tables\_S1\_S5.pdf - Supplementary Data
