# Supplementary material for: Pepper CabZIP63 acts as a positive regulator during Ralstonia solanacearum or high temperature–high humidity challenge in a positive feedback loop with CaWRKY40
Source: J Exp Bot. 2016 Mar 1;67(8):2439–51. doi: 10.1093/jxb/erw069 (PMC4809298; doi:10.1093/jxb/erw069)
Supplement: Supplementary Data [file supp_erw069_supplementary_figures_S1_S6_tables_S1_S5.pdf]

Pepper CabZIP63 acts as a positive regulator during Ralstonia solanacearum or high-temperature-high-humidity challenge in a positive feedback loop with CaWRKY40

Lei Shen[1,2,#], Zhiqin Liu[1,2, #], Sheng Yang[1,2, #], Tong Yang[1,3], Jiaqi Liang[1,3], Jiayu Wen[1,2], Yanyan Liu[1,2], Jiazhi Li[1,3], Lanping Shi[1,2], Qian Tang[1,2],Wei Shi[1,2], Jiong Hu[1,2], Cailing Liu[1,2], Yangwen Zhang[1,2], Wei Lin[1,2], Rongzhang Wang[1,2], Huanxin Yu[1,2], Shaoliang Mou[1,3], Ansar hussain[1,2], Wei Cheng[1,2], Hanyang Cai[1,2], Li He[4], Deyi Guan[1,2], Yang Wu[4,]* and Shuilin He[1,2,]*

Supplementary Data

Fig. S1

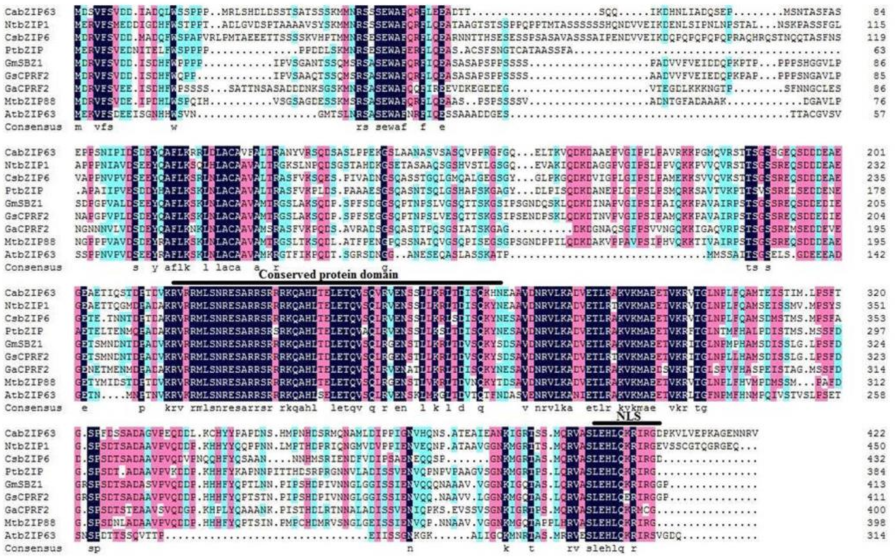

Fig. S1. Comparison of amino acid sequences deduced from pepper *CabZIP63*with the representative closely related proteins from other plant species including *NtbZIP1* (*Nicotianatabacum*, AY061648.1), *CsbZIP6* (*Camellia sinensis*, KC008716.1), *PtPOPTR_0013s03800g* (*Populus trichocarpa*, XM_002319036.2), *Gm*SBZ1 (*Glycine max*, NM_001251024), *MtbZIP88* (*Medicago truncatula*, KEH43021), *GsCPRF2* (*Glycine soja*, KHN04932.1), *GaCPRF2* (*Gossypium arboretum*, KHG10545.1) and *AtbZIP63* (*Arabidopsis thaliana*, NP_568508.2). Green shading, 50%-75% identity; red shading, 75%-100% identity; black shading, 100% identity. The alignment was carried out using DNAMAN5 (Lynnon Biosoft, USA).

Fig. S2

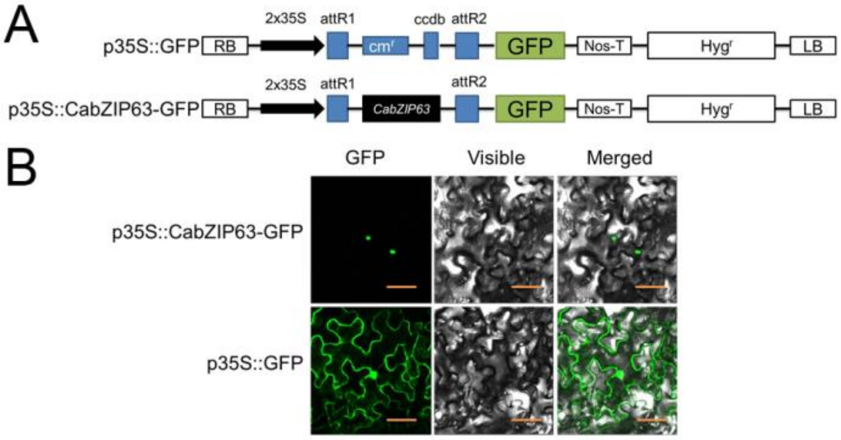

Fig. S2. Subcellular localization of CabZIP63 in *N. benthamiana* leaves using an *Agrobacterium*-infiltration based approach. **(A)** Schematic representation of *35S::CabZIP63-GFP* and *35S::GFP* constructs. **(B)** CabZIP63-GFP exclusively localized in the nucleus of *N. benthamiana* leaves; green fluorescent protein (GFP) localized throughout the whole cells. Bars = 50 μm. All images were taken by Leica confocal microscopy at 48 hours after agro-infiltration.

Fig. S3

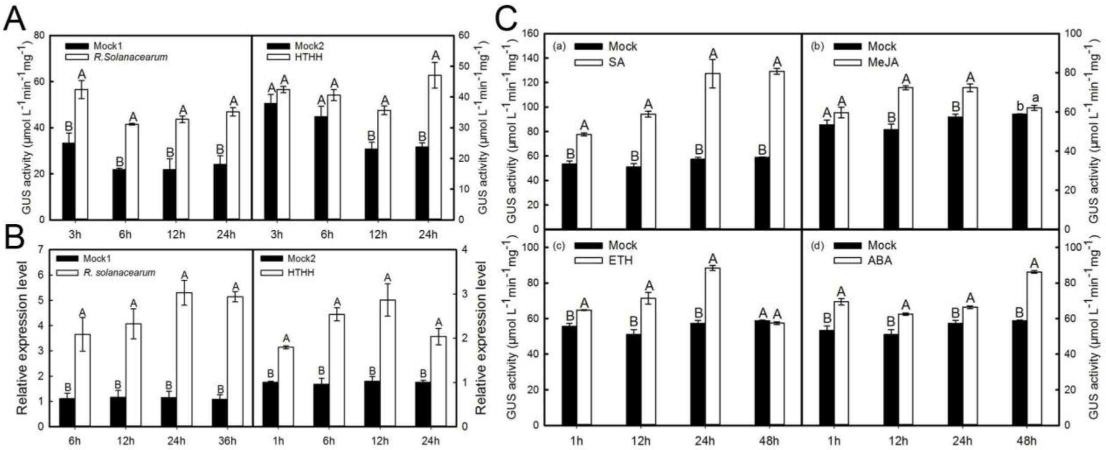

Fig. S3. The expression of *CabZIP63* was induced by RSI and HTHH as well as exogenous applied SA, MeJA, ETH or ABA. **(A)** The promoter fragment upstream the translation start code with 2000 bps in length was cloned into the vector of pDMC163 upstream GUS reporter gene and the generated *pCabZIP63::GUS* was transformed into *Agrobacterium* GV3101, which was further infiltrated into pepper leaves. At 24 hpi, the infiltrated leaves were further inoculated with *R. solanacearum* strain FJC100301, or treated with 38 ℃ under 90% of humidity, and the leaves were harvested at appropriate time points for GUS expression assay. Mock1 was the treatment of $MgCl_2$ and Mock2 was the treatment of room temperature (25 ℃) under 50% of humidity. **(B)** The transcript levels of *CabZIP63* measured at different time points in pepper leaves after RS inoculation and HTHH treatment, respectively, by real-time RT-PCR. **(C)** The expression of GUS driven by *pCabZIP63* was promoted by exogenous application of SA, MeJA, ETH or ABA. The leaves of pepper plants at 8-leaf stage ware infiltrated with GV3101 cells (OD_{600} = 0.6) containing *pCabZIP63::GUS*, at 24 hours later, the plants were sprayed with 1 mm SA (a), 100μm MeJA (b), 100μm ETH (c), or 100μm ABA (d), and the leaves were harvested at different time points for GUS activity assay by microplate reader using pepper leaves treated with ddH_2O as mock. Data represent the means ± SD from four independent biological replicates. Different capital letters indicate significant differences from four independent biological replicates based on the LSD test (P<0.01). Different lower-case letters indicate significant differences from three

independent experiments based on the LSD test (P<0.05).

Fig. S4

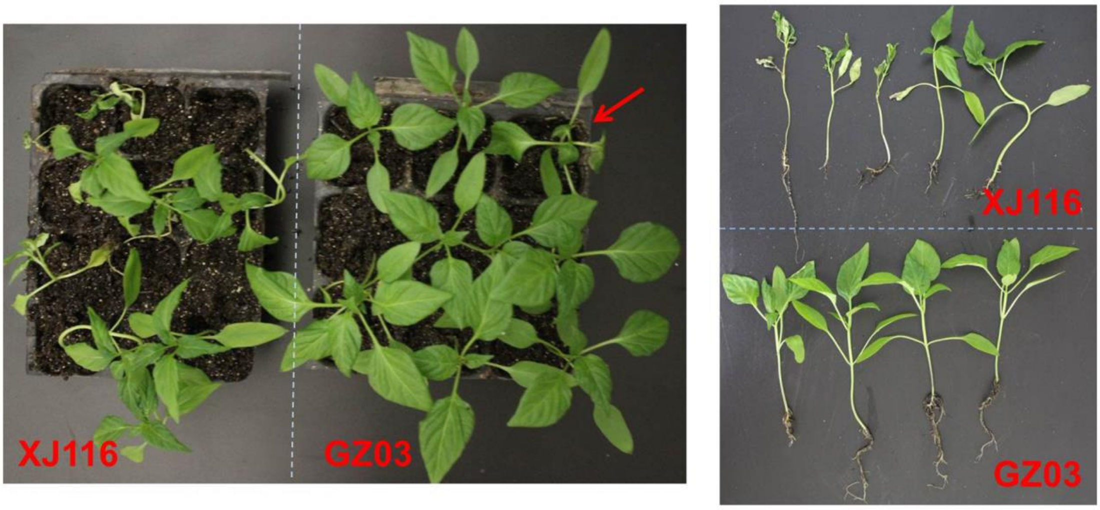

Fig. S4. The detection of virulence of *R. solanacearum* strain FJC100301 by root-irrigation. GZ03, an inbred line with middle level of resistance to *R. solanacearum*, and XJ116, another inbred line susceptible to *R. solanacearum* were used. Each pot containing one plant at of 6-8 leaf stage with its root injured was irrigated with 5ml FJC100301 suspension containing 1×10^5 cells per milliliter, and then the pots were kept in a growth room under a condition of 28 ℃ and soil moisture was kept more than 90%. The result showed that 10 of the 12 XJ116 plants exhibited disease symptom, but only one of the 9 GZ03 plants exhibited faint blight symptom.

Fig. S5

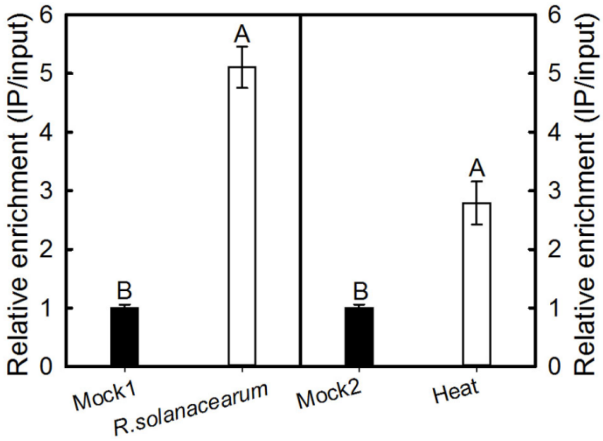

Fig. S5. The binding of CabZIP63 to p*CaWRKY40* was promoted by RSI and HTHH. GV3101 cells containing *35S::CabZIP63-HA* was infiltrated into pepper leaves, and the pepper plants were inoculated with *R. solanacearum* or treated with 38 ℃ under 90% of humidity at 24 hpt. At 48 hours later, the infiltrated leaves were harvested for chromatin preparation. Relative DNA amount pulled by CabZIP63-HA with the treatment of RSI and HTHH in pepper leaves were determined using real-time quantitative PCR analysis. Mock1 was the treatment of $MgCl_2$ and Mock2 was the treatment of room temperature (25 ℃) under 50% of humidity. Relative enrichment levels of samples of $MgCl_2$ or room temperature were set to 1 after normalization by input. Data represent the means ± SD from four independent biological replicates. Different upper-case letters indicate significantly different means, as analyzed by Fisher's protected LSD test (P<0.01).

Fig. S6

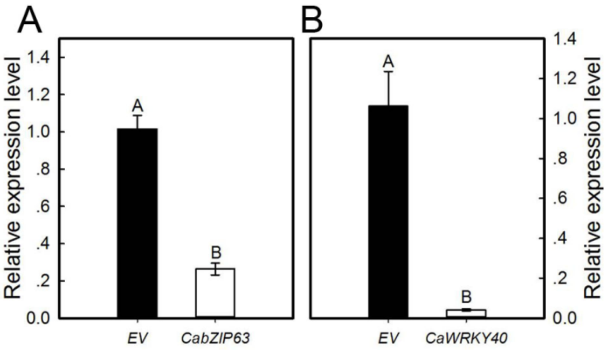

Fig. S6. Relative expression level of endogenous *CabZIP63* or *CaWRKY40* in *CabZIP63-SRDX* or *CaWRKY40-SRDX* transiently overexpressing pepper leaves by real-time RT-PCR. **(A)** Relative expression level of *CabZIP63* in *CabZIP63-SRDX* transiently overexpressing pepper leaves by real-time RT-PCR using the specific primers designed according to the 3’ UTR of *CabZIP63*. **(B)** Relative expression level of *CaWRKY40* in *CaWRKY40-SRDX* transiently overexpressing pepper leaves by real-time RT-PCR using the specific primers designed according to the 3’ UTR of *CaWRKY40*. Data represent the means ± SD from four independent experiments. Different letters indicate significant differences, as determined by Fisher’s protected LSD test (P<0.01).

Table S1. Grading standards for evaluation of disease resistance of pepper plant to *R. solanacearum* by root-irrigation.

Disease level	Symptom Descriptions
0	No symptoms
1	The roots of asymptomatic; not wilting or a small amount of restorable wilting of leaves
2	1cm-2cm constriction of seedling root; non-recoverable wilting of leaves
3	The constriction of seedling root more than 2 cm; obvious wilting or abscission of leaves
4	Constriction of seedling root; abscission of all leaves except as meristem or plant wilting
5	Plants withered

Table S2. Pepper primers used for vectors construction in this study.

Gene	Forward primers (5' to 3')	Reveres primers (5' to 3')	Size (bp)
CabZIP63[1]	GGGGACAAGTTTGTACAAAAAAGCAGGCTT<br>CATGGATAGCGTGTTTTCGGTG	GGGGACCACTTTGTACAAGAAAGCTGGGTCTT<br>AAGTCACTCTATTGTTCTC	1272
CabZIP63[2]	GGGGACAAGTTTGTACAAAAAAGCAGGCTT<br>CATGGATAGCGTGTTTTCGGTG	GGGGACCACTTTGTACAAGAAAGCTGGGTCAG<br>TCACTCTATTGTTCTCCCC	1269
CabZIP63[3]	GGGGACAAGTTTGTACAAAAAAGCAGGCTT<br>CATGGATAGCGTGTTTTCGGTG	GGGGACCACTTTGTACAAGAAAGCTGGGTCTT<br>AAGCGAAACCCAAACGGAGTTCTAGATCCAGA<br>TCGAGAGTCACTCTATTGTTCTCCCC	1338
CabZIP63[4]	GGGGACAAGTTTGTACAAAAAAGCAGGCTT<br>CCAACATTCATCTGTCAACTC	GGGGACCACTTTGTACAAGAAAGCTGGGTCTC<br>TTTCTCTCTCCTCCCTAG	2000
CabZIP63[5]	GGGGACAAGTTTGTACAAAAAAGCAGGCTT<br>CAAGGAAGATTTCGCAGAC	GGGGACCACTTTGTACAAGAAAGCTGGGTCCA<br>AAGGGGAAAAGAGTGA	229

[1] Primers for full-length *CabZIP63* cloning.

[2] Primers for the vector constructing of *35S::CabZIP63-83*.

[3] Primers for *CabZIP63-SRDX* cloning.

[4] Primers for full-length *CabZIP63* promoter cloning.

[5] Primers for the vector constructing of TRV::*CabZIP63*.

Table S3. Pepper primers used for real-time RT-PCR in this study.

Gene	Accession no.	Forward primers (5' to 3')	Reveres primers (5' to 3')	Size (bps)
CabZIP63[a]		AAGGAAGATTTCGCAGAC	CAAAGGGGAAAAGAGTGA	229
CabZIP6[b]		ACGACATTGCCGATCAATTA	GCAAACGATGCGGTATTAGA	226
CabZIP6[c]		TTAGATATCCCTCCTATAGG	ACGGAGTTCTAGATCCAGATC	207
CaWRKY40[d]	AAX20040.1	AAGTCCAGCAGAGCAGTCAA	AACAATTGTCTAAGCCATCCG	152
CaWRKY40[e]	AAX20040.1	GGTGTGGCAGATGATAGTGC	CCAGGCACAACATCCAAGT	122
CaWRKY40[f]	AAX20040.1	CAGAGTGCTAGTCCTACCGGT	ACGGAGTTCTAGATCCAGATC	219
CaPR1	AF348141.1	GCCGTGAAGATGTGGGTCAATGA	TGAGTTACGCCAGACTACCTGAGTA	108
CaNPR1	X61679.1	ACTTCTTCGCCGACGCCAAG	GCCAACACATTCACCAGAGCATC	190
CaDEF1	AF442388	GTGAGGAAGAAGTTTGAAAGAAAGTAC	TGCACAGCACTATCATTGCATACAATTC	267
CaABR1	CA524559	ATGACAGGCACAACAGAAGAAAAT	AATAAGTTATGACAGAGCCATTTT	148
CaHSP24	HM132040	GTTCGTCTAGCAGTTTGGTTCGGTTG	GTAATTTAACTAAACAGACTCTTACAACC	152
CaACTIN	GQ339766	AGGGATGGGTCAAAAGGATGC	GAGACAACACCGCCTGAATAGC	225
18s rRNA	EF564281	CCGGTCCGCCTATGGTGTGCACCGGTCGTC	GCAGTTGTTCGTCTTTCATAAATCCAAGAA	285

[a]Specific primers for detecting relative expression of *CabZIP63* designed according to of the sequence in3' UTR.

[b]Specific primers for detecting relative expression of *CabZIP63* designed according to of the sequence in ORF domain.

[c]Specific primers for detecting relative expression of *CabZIP63* designed according to of the sequencein ORF (Forward primer) and SRDX (Reveres primers) domain.

[d]Specific primers for detecting relative expression of *CaWRKY40* designed according to of the sequence in3' UTR.

[e]Specific primers for detecting relative expression of *CaWRKY40* designed according to of the sequence in ORF domain.

[f]Specific primers for detecting relative expression of *CaWRKY40* designed according to of the sequence in ORF (Forward primer) and SRDX (Reveres primers) domain.

Table S4. Pepper primers used for ChIP PCR or real-time RT-PCR in this study.

Primer name	Forward primers (5' to 3')	Reveres primers (5' to 3')
ATTB	GGGGACAAGTTTGTACAAAAAAGCAGGCTTC	GGGGACCACTTTGTACAAGAAAGCTGGGTC
pCaWRKY40 C-box	TATTCTCAAAAAATTCAATC	ATTCAAGTGTTTGTTTACAA
pCaWRKY40 G-box	AACCAAGATTGTACTATAGC	AATTGCCCTTTTAAGAAGAG
pCaWRKY40 mock[b]	TGCGGAAGCTATGTAGACCA	TTTGATTCTGCTTGTTGATA
pCabZIP63 1 G-box	TTTTATCAAACTTTAAAAGAAGAT	ACATCTATGCTCCTATGGGATGGT
pCabZIP63 2 G-box	TACAACAAAATAATACAATACTG	ATTGGGGAAATATGATTCATGTT
pCabZIP63 mock[a]	GAATAGTACTCCTAAATATG	GATGAGCATCAATCATATTC
pCaPR1 1W-box	AGCTCCATCCCAAACCAACC	TGGTGTTGGGTCTGTGAGGC
pCaNPR1 1W-box	ATTTCCTGCATTAGTTA	TGAAAAGAGTGACACCG
pCaDEF1 1W-box	AATCAGTGCCGACTGTGGGG	GCGCACCTCGGCGCTGAGCT
pCaHSP24 1W-box	TTTGACTCAATTTACTAGCG	CGTAAACTATTGATATTTTA

Table S5. The plant numbers with different level of disease resistance in VIGS assay of *CabZIP63* against *R. solanacearum* strain FJC100301 infection.

Plant type	infection grade						average disease index	Plant total
	0	1	2	3	4	5		
TRV::*CabZIP63*	3	12	8	10	11	16	3.0	60
TRV::*00*	16	22	19	1	1	1	1.2	60
